# Prevalence and Characterisation of Antimicrobial Resistance, Virulence Factors and Multilocus Sequence Typing (MLST) of *Escherichia coli* Isolated from Broiler Caeca

**DOI:** 10.3390/ani15101353

**Published:** 2025-05-08

**Authors:** Ah-Ran Lee, Martin John Woodward, Caroline Rymer

**Affiliations:** 1 Animal Resources Research Center, Konkuk University, 120 Neungdong-ro, Gwangjin-gu, Seoul 05029, Republic of Korea; 2 Department of Food and Nutritional Sciences, School of Chemistry, Food and Pharmacy, University of Reading, Whiteknights, Reading RG6 6DZ, UK; m.j.woodward@reading.ac.uk; 3 Folium Science, St Philips Central, Albert Road, Bristol BS2 0XJ, UK; 4 Department of Animal Sciences, School of Agriculture, Policy and Development, University of Reading, Whiteknights, P.O. Box 237, Reading RG6 6EU, UK; c.rymer@reading.ac.uk

**Keywords:** broiler, *Escherichia coli*, *Lactococcus lactis*, *Limosilactobacillus fermentum*, virulence gene

## Abstract

This study was undertaken to determine the effect of bird age and administering either *Lactococcus lactis* ssp. lactis 1 (LL) or *Limosilactobacillus fermentum* 1 (LF) in the drinking water on the prevalence of antimicrobial resistance by phenotypic test, multilocus sequence typing (MLST) and virulence genes of *Escherichia coli* (*E. coli*) isolated from broiler caeca by whole-genome sequencing (WGS) analysis. Male (Ross 308) day-old chicks (240) were reared for 28 days. Water was provided either untreated (CON) or with LL (10^7^/mL) or LF (10^7^/mL) via a nipple drinker on three days each week during the starter phase (days 1, 3, 5, 7, 9 and 11 d) in eight replicate pens per treatment, with initially ten chicks per pen. One chick from each pen was sacrificed when LL or LF was added to the water, and again on d 14 and 28. There was no evidence that LL and LF had any effect on the prevalence of antimicrobial resistance and virulence genes in *E. coli* isolates. The population density of *Lactobacillus* sp. and coliforms decreased with age (*p* < 0.001). The high resistance of *E. coli* to ampicillin and tetracycline was maintained throughout the life of the broilers. The prevalence of virulence genes was greatest during the starter phase but declined when birds were 28 days of age (*p* < 0.05). In birds < 14 d of age, *E. coli* MLST 457, 1640, 1485 and 155 were dominant, and these carried *iuc*D, *irp*2, *ast*A, *iut*A and *iro*N genes. When birds were 28 d of age, MLST 1286, 1112 and 973 predominated, and these carried few virulence genes. This suggests that young birds were more susceptible to putative pathogenic *E. coli* than older birds. Supporting the development of a healthy microbiome that might control the proliferation of potentially pathogenic *E. coli* is an area of future research.

## Simple Summary

This study investigated the effect of broiler age and the addition of beneficial bacteria to drinking water on the presence of potentially harmful and antibiotic-resistant *Escherichia coli* in the gut. A total of 240 male chicks were reared for 28 days. During early growth, some birds were given water supplemented with either *Lactococcus lactis* or *Limosilactobacillus fermentum*, while others were given plain water. Samples were taken at different ages to monitor changes in gut bacteria. The results showed that adding these beneficial bacteria did not reduce the number of potentially harmful or antibiotic-resistant *Escherichia coli*. However, the type of bacteria in the gut changed as the birds aged. Younger birds had more potentially harmful strains of *Escherichia coli*, while older birds had less harmful types. This suggests that very young chickens may be more susceptible to infection. This study highlights the importance of promoting healthy gut bacteria early in life to protect poultry from disease. These findings may contribute to future strategies that aim to promote the natural health of the gut in animals.

## 1. Introduction

Most *E. coli* strains are commensal and are adapted to survive in the intestine, and *E. coli* are regularly studied as a representative indicator of AMR and virulence [1,2]. Specific strains of antimicrobial-resistant (AMR) *E. coli* carry virulence factors and may lead to a variety of infections in humans and in animals [3,4]. Many virulence-associated genes (VAGs) have been associated with infection and colibacillosis in poultry [5,6,7]. A variety of VAGs have also been implicated in causing extraintestinal diseases such as septicaemia, air sacculitis, and cellulitis, peritonitis and salpingitis in birds [8,9].

Virulence genes are generally located in mobile factors such as plasmids, allowing them to transfer to other (formerly) commensal *E. coli* [10]. Reducing any selective advantage conferred by these virulence factors by enabling the establishment of more benign serotypes (perhaps through dietary intervention to alter the gut environment) may be a means of reducing the spread of pathogenic *E. coli* if these are transferred with the virulence genes. One such way of addressing losses from infection with potentially AMR and pathogenic *E. coli* may be by using feed additives that may competitively exclude by niche blocking, alter the supply of nutrients in the gut, alter the gut environment or alter the composition of the gut microbiome.

There is considerable evidence that *Lactococcus lactis* and *Limosilactobacillus fermentum* have beneficial properties associated with the prevention of pathogen growth and maintaining a healthier gut microbiome [11,12,13]. In preliminary in vitro tests conducted in our laboratory (unpublished), two species of lactic acid bacteria—*Lactococcus lactis* (LL), isolated from the chicken caecum, and *Limosilactobacillus fermentum* (LF), isolated from the pig intestine—were identified by NMR analysis to produce higher levels of lactic acid and ethanol, respectively. Both strains exhibited strong inhibitory effects on the growth of *Escherichia coli*.

In a previous bird study [14], it was observed that the birds were most susceptible to colonisation with coliforms carrying AMR and VAGs at the end of the starter phase (around 8 d old). The AMR and virulence of *E. coli* in the chicken caecum then declined through the grower and finisher phases. Therefore, to confirm the findings of the previous study and determine exactly when the birds were at their most vulnerable, this research focused on the starter phase, monitoring changes in AMR and VAG profiles of *E. coli* and determining the effect of administering lactic acid bacteria (LAB) in the starter phase. It was hypothesised that an intervention with lactic acid bacteria might alter the AMR and virulent *E. coli* status in young birds by advancing the maturation of the microbiome.

The impact of bird age and LAB administration on the detection of phenotypic AMR and VAGs in *E. coli* isolates may be investigated by whole-genome sequencing (WGS) analysis. Furthermore, to better characterise the *E. coli* isolated from the caecum, this can be used to analyse the diversity and evolution of the *E. coli* and identify the relationship between certain *E. coli* sequence types and the carriage of virulence genes. Accordingly, the objective of this experiment was to determine the effect of bird age and administering either *Lactococcus lactis* or *L. fermentum* in the drinking water of broiler chicks during their starter phase (1–11 d of life) on the prevalence of AMR and virulence genes in *E. coli* isolated from the birds’ caeca.

## 2. Materials and Methods

All birds were housed at the Centre for Dairy Research (CEDAR), University of Reading, Reading, UK. Chickens were kept according to the Code of Recommendations for the Welfare of Meat Chickens and Breeding Chickens [15] and the Ross 308 broiler management handbook [16]. This study was approved by the Ethical Committee of the University of Reading, Reading, UK (Approval No. DASPGR 2024_ARL), with all procedures following institutional and national guidelines including the Animals (Scientific Procedures) Act 2013 (UK).

### 2.1. Experimental Design, Animals and Diet

A total of 240 chicks (day-old, male Ross 308, purchased from PD Hook, Cote, Oxfordshire, UK) were used in a 28-day feeding trial. All chicks were weighed on arrival, wing tagged, blocked by liveweight, and then randomly placed in 1 of 24 cages (ten chicks per cage) and fed a common starter diet until the birds were 14 days of age. *Lactococcus lactis* ssp. *lactis* 1 (isolated from a chicken in the previous bird study) and *Limosilactobacillus fermentum* 1 (isolated from a pig; University of Surrey, Guildford, UK) were administered via the drinking water. The identification of these strains was confirmed using the analytical profile index (API) test. Stock cultures of each isolate were incubated overnight in De Man, Rogosa and Sharpe (MRS) broth. Population density was then estimated by serial dilution and measurement of the optical density of the incubation medium at 600 nm. Cultures were then centrifuged at 3354× *g* for 5 min (Eppendorf centrifuge 5804R, Hamburg, Germany) and washed with sterile distilled water. An appropriate volume of the culture was then added to a measured volume of the drinking water in the hopper to attain the desired final concentration of lactic acid bacteria. Water was therefore provided either untreated (Control) or with a preparation of *Lactococcus lactis* (LL; 10^7^ CFU/mL water offered) or *L. fermentum* (LF; 10^7^ CFU/mL water offered) via a nipple drinker on three days each week during the starter phase (1, 3, 5, 7, 9 and 11 d). There were eight replicate cages for each of these experimental treatments. The nipple drinker was replenished with fresh (untreated) water when required.

On day 14, all birds were weighed and then transferred to a floor pen (four birds per pen, all birds in each pen originated from the same cage), with eight replicate pens per treatment. All birds were then fed a common grower/finisher diet. The ingredient and nutrient composition of the diets is shown in Table 1. The diets were fed in the form of a mash and were manufactured by Target Feeds (Whitchurch, UK). Samples of each diet were analysed for starch, sugars, oil, crude protein and amino acids (Sciantec, York, UK). Feed and water were provided for ad libitum consumption. Lighting was provided via incandescent lights with 23 h continuous light per 24 h period for the first seven days, followed by 18 h continuous light (6 h darkness) in each 24 h period. The birds were brooded according to the breeder’s recommendations using infrared lights to provide supplementary heat when necessary.

### 2.2. Bird Performance

Birds were weighed individually on days 1, 14 and 28. Mean body weight increase was then calculated on a pen basis during the starter and grower/finisher periods. Feed intake was determined and FCR calculated on a pen basis during the grower/finisher period.

### 2.3. Sample Collection

On day 1, a total of 24 birds were randomly selected and sacrificed by cervical dislocation. The contents of whole intestinal tract (from proximal duodenum to ileo-caecal junction) and the yolk sac were taken and placed on ice for storage and transportation. The yolk sac was included to assess the potential vertical transmission of antibiotic-resistant *Escherichia coli* from breeder hens or through egg contents. These were analysed for the determination of the population size (CFU/g) of lactic acid bacteria and coliforms, and for the determination of antimicrobial resistance within 5 h of collection. Coliforms were then further analysed by whole-genome sequencing. On days 3, 5, 7, 9, 11 and 14, one bird from each cage (total of 24 cages) was selected randomly and sacrificed. Digesta from the caecum were taken and placed on ice for transportation, and these were analysed for the same determination as above. After the birds were allocated to their grower/finisher pens, one bird from each pen was randomly selected and sacrificed on day 28. Samples of digesta from the caecum were again taken and analysed as before.

### 2.4. Determination of Viable E. coli and Lactic Acid Bacteria Populations and Isolation of E. coli

The samples of whole gut digesta and yolk sac (from day-old chicks) and the samples of caecal digesta (taken from the older birds) were analysed for the determination of the population size (CFU/g) of lactic acid bacteria and coliforms. The samples were weighed and serially diluted with phosphate-buffered saline (PBS, 0.01 M), and 100 µL of the suspension was then spread on De Man, Rogosa and Sharpe (MRS) agar (for the enumeration of lactic acid bacteria) or MacConkey agar (for the enumeration of coliforms, in caecal digesta samples only). Plates were incubated (37 °C, 48 h) in an anaerobic incubator (Whitley MG1000 anaerobic workstation) for the lactic acid bacteria cultures and aerobically incubated overnight at 37 °C for the coliforms. Colonies were then counted (Gallenkamp Colony Counter CNW 325 030Y, Hailsham, UK) to determine population sizes (CFU/g). A single colony of *E. coli* was selected at random from each MacConkey plate (eight replicate plates per treatment, per sampling time) and transferred to a separate, sterile microcentrifuge tube containing 500 µL of nutrient broth. These cultures were incubated at 37 °C for 18–24 h. We transferred 100 µL of the resulting *E. coli* suspension to a cryovial (Mast group, Mastdisks, Merseyside, UK) and stored it at −80 °C pending further analysis.

### 2.5. Determination of Antimicrobial Resistance

Resistance to antimicrobials by the colonies of *E. coli* grown on MacConkey agar plates was determined by replicate plating using plates of MacConkey agar prepared with different antibiotics (ampicillin, nalidixic acid, tetracycline and chloramphenicol, each at a concentration of 20 µg/mL). These antibiotics are also commonly used for susceptibility testing in *E. coli*. Plates were then incubated (37 °C, 18–24 h), and growth of colonies on each plate was then determined. When growth was observed on plates containing antibiotics, it was deemed that the colony on the parent plate was resistant to that antibiotic. The proportion of AMR was estimated from the number of colonies grown on the plate containing antibiotic as a proportion of the number of colonies grown on the parent plate.

### 2.6. Extraction of Genomic DNA

The genomic DNA from total 68 isolates of *E. coli* was extracted to determine the presence of *E. coli* virulence factors. DNA was extracted from seven yolk sacs collected on day 1 and from 61 caecal digesta samples. The caecal digesta samples consisted of seven samples from day 1 and nine digesta samples (one from each of three cages per treatment) on days 3, 5, 7, 11, 14 and 28. The *E. coli* isolates with multidrug resistance were selected for DNA extraction and WGS analysis. Genomic DNA was extracted using Pure-gene yeast/bact Kit B (Qiagen, Venlo, Netherlands), and standard protocols for fresh samples of Gram-negative bacterial cultures were followed, as described below and by Lee, Aldeieg, Woodward, Juniper and Rymer [14]. Isolated cultures of *E. coli* were streaked on Luria–Bertani (LB) agar vegitone plates and incubated at 37 °C overnight. Colonies of *E. coli* were then taken from these plates and transferred to a 1.5 mL sterile microcentrifuge tube. These were then incubated at 80 °C for 5 min after adding 300 µL of cell lysis solution to the pellet. The tubes were put on ice for 20 min. We added 1.5 µL of RNase A solution and mixed by inverting 25 times. Next, 100 µL of protein precipitation solution was added and vortexed vigorously for 20 s at high speed. The mixture was then centrifuged at 12,045× *g* for 3 min (Eppendorf minispin, Hamburg, Germany). The supernatant was transferred to a clean 1.5 mL microcentrifuge containing about 700 µL of 99.5% ethanol and mixed by gently inverting 50 times. The mixture was centrifuged at 12,045× *g* for 1 min, and the supernatant was carefully discarded. A total of 300 µL of 70% ethanol was added to the DNA pellet and inverted several times. The mixture was then centrifuged at 12,045× *g* for 1 min, and again the supernatant was discarded and the pellet was allowed to air dry for 5 min. We added 100 µL of DNA hydration solution, and the mixture was vortexed for 5 s, then incubated at 65 °C for 1 h, followed by incubation overnight at room temperature with gentle shaking. The purification of DNA was determined with a Nanodrop spectrophotometer (ND 2000, Thermo Fisher Scientific, Waltham, MA, USA). The quality of DNA was evaluated according to the method of Tonks [17]. The Nanodrop tube was cleaned by pipetting 1.5 µL of distilled water onto it and wiping with a medical wipe. The Nanodrop was blanked with 1.5 µL of distilled water. Next, 1.5 µL DNA solution was added to the measuring stage, the DNA concentration was recorded in ng/µL, and the 260:280 nm ratio was also recorded (1.82 ± 0.07 was required). DNA stock solutions were stored at −20 °C pending the analysis of virulence genes by WGS.

### 2.7. Whole-Genome Sequencing (WGS) and Data Analysis

All extracts were submitted to the Quadram Institute of Bioscience to determine the genotype of individual *E. coli* isolates by WGS. Genomic DNA was normalised to 0.5 ng/µL with EB (10 mM Tris-HCl). Then, 0.9 µL of TD Tagment DNA Buffer (Illumina Catalogue No. 15027866, San Diego, CA, USA) was mixed with 0.09 µL TDE1, Tagment DNA Enzyme (Illumina Catalogue No. 15027865, San Diego, CA, USA) and 2.01 µL PCR-grade water in a master mix and 3 µL added to a chilled 96-well plate. Following that, 2 µL of normalised DNA (1 ng total) was pipette mixed with the 3 µL of the Tagmentation mix and heated to 55 °C for 10 min in a PCR block. A PCR master mix was made up using 4 µL kapa2G buffer, 0.4 µL dNTPs, 0.08 µL polymerase and 6.52 µL PCR-grade water, contained in the Kap2G Robust PCR kit (Sigma Catalogue No. KK5005, St. Louis, MI, USA), per sample and 11 µL added to each well to be used in a 96-well plate. After that, 2 µL each of P7 and P5 of Nextera XT Index Kit v2 index primers (Illumina Catalogue No. FC-131-2001 to 2004, San Diego, CA, USA) was added to each well. Finally, 5 µL of Tagmentation mix was added and mixed. The PCR was run at 72 °C for 3 min, 95 °C for 1 min, 14 cycles of 95 °C for 10 s, 55 °C for 20 s and 72 °C for 3 min. Following the PCR reaction, the libraries were quantified using the Promega QuantiFluor^®^ dsDNA System (Promega Corporation, Madison, WI, USA) (Catalogue No. E2670) and run on a GloMax^®^ Discover Microplate Reader (Promega Corporation, Madison, WI, USA). Libraries were pooled following quantification in equal quantities. The final pool was double-SPRI size selected between 0.5 and 0.7× bead volumes using KAPA Pure Beads (Roche Catalogue No. 07983298001, Basel, Switzerland). The final pool was quantified on a Qubit 3.0 instrument and run on a D5000 ScreenTape (Agilent Catalogue No. 5067-5579, Santa Clara, CA, USA) using the Agilent Taestation 4200 to calculate the final library pool molarity. The pool was run at a final concentration of 1.5 pM on an Illumina Nextseq500 instrument using a Mid Output Flowcell (NSQ^®^ 500 Mid Output KT v2(300 CYS) Illumina Catalogue FC-404-2003, San Diego, CA, USA) following the Illumina recommended denaturation and loading recommendations, which included a 1% PhiX spike (PhiX Control v3 Illumina Catalogue FC-110-3001, San Diego, CA, USA). Data were uploaded to Basespace (www.basespace.illumina.com; URL accessed on 11 December 2019), where the raw data were converted to eight FASTQ files for each sample. Using Bracken, all samples were reported as having 90%+ reads matching to an *E. coli* reference genome. ECtyper was used to obtain the O and H serotypes for the *E. coli* strains. The virulence genes were also identified using ARIBA and the VFDB full database.

### 2.8. Statistical Analysis

The effect of the administration of either LL or LF on bird performance (body weight gain, feed intake, feed conversion ratio) was determined by analysis of variance using the general linear model of Minitab (Minitab 19, Minitab Inc., State College, PA, USA). The effect of bird age and administration of lactic acid bacteria on the population density of *Lactobacillus* spp. and coliforms, and the proportion of phenotypic antimicrobial resistance was determined by ANOVA (mixed-effects model). Fixed factors were bird age and administration of LAB, and pen was a random factor. Tukey’s post hoc test was used to compare means to observe significance at the level of *p* < 0.05. The association between bird age and *E. coli* MLST was determined by Chi-square analysis. Associations between the carriage of virulence genes and MLST *E. coli* were determined by Chi-square analysis.

## 3. Results

### 3.1. Growth Performance and Population Size of Coliforms and Lactic Acid Bacteria in the Caecum

**Figure 1:**
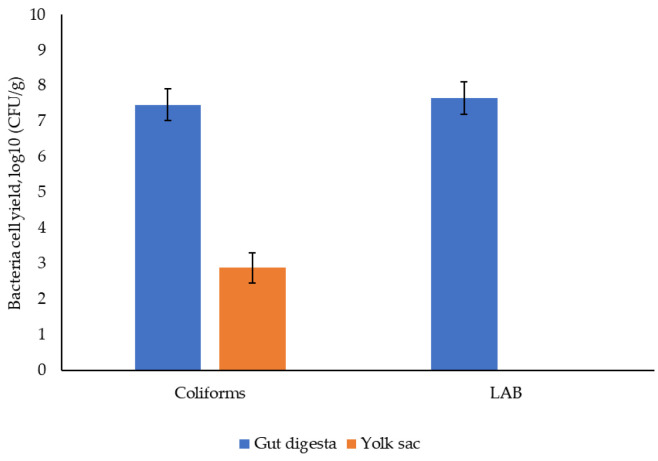
Population density of total coliforms and lactic acid bacteria (LAB) in the gut digesta and yolk sac of day-old chicks.

**Figure 2:**
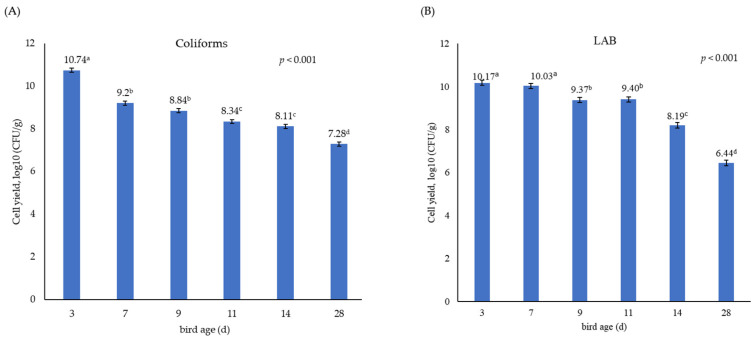
Effect of bird age on the population density of bacteria. (**A**) Coliforms and (**B**) lactic acid bacteria (LAB). Data are presented as mean ± standard error. Values with different superscripts (a–d) are significantly different (*p* < 0.001).

Table 2 shows that the administration of either LL or LF had no significant effect on the growth performance of broiler chickens. Feed intake was close to that expected for birds of this age, but growth was slower and FCR consequently higher. This suggests that the birds in this study, while not displaying any clinical signs, were of sub-optimal health. Even as day-old chicks, there was a large population in the gut of both coliforms (7.47 log10 CFU/g) and lactic acid bacteria (7.66 log10 CFU/g), which was larger than the population observed in the yolk sac (log10 CFU/g of 2.88) for *E. coli* (Figure 1). The LAB population in the yolk sac was not analysed because of the focus on the *E. coli* population and its virulence. There was no significant difference between treatments in the overall population of coliforms and lactic acid bacteria in caecal samples taken throughout the birds’ life. The population density of coliforms (from 10.74 to 7.28 log10 CFU/g) and LAB (from 10.17 to 6.44 log10 CFU/g) declined between day 3 and day 28 of bird age (*p* < 0.001, Figure 2).

### 3.2. Antimicrobial Resistance of E. coli in Caecal Digesta

**Figure 3:**
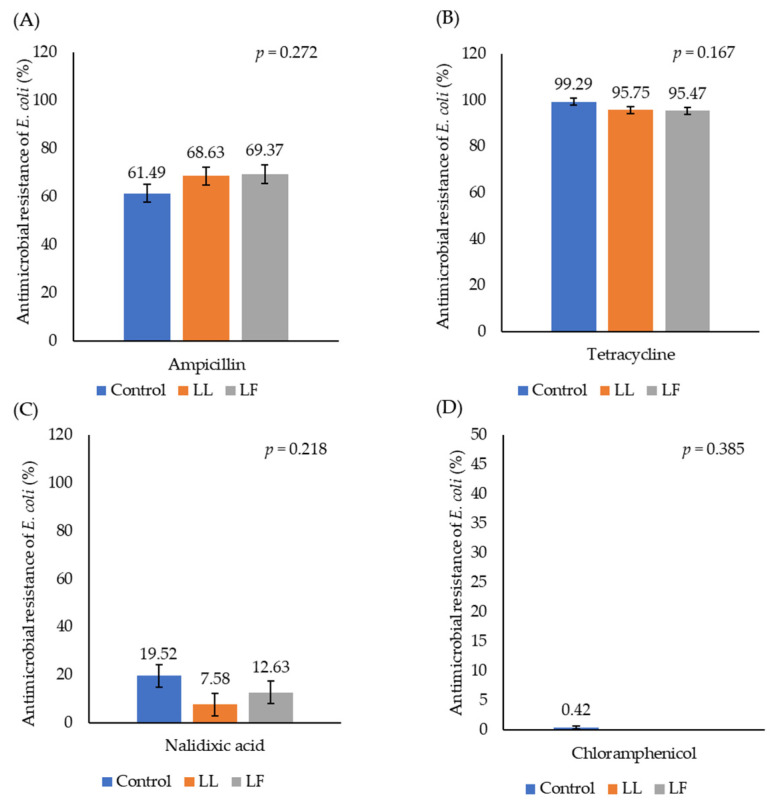
The effect of administration of either *Lactococcus lactis* (LL) or *Lactobacillus fermentum* (LF) on the proportion of isolates of *E. coli* that were resistant to different antimicrobials (20 µg/mL). (**A**) ampicillin, (**B**) tetracycline, (**C**) nalidixic acid, and (**D**) chloramphenicol.

**Figure 4:**
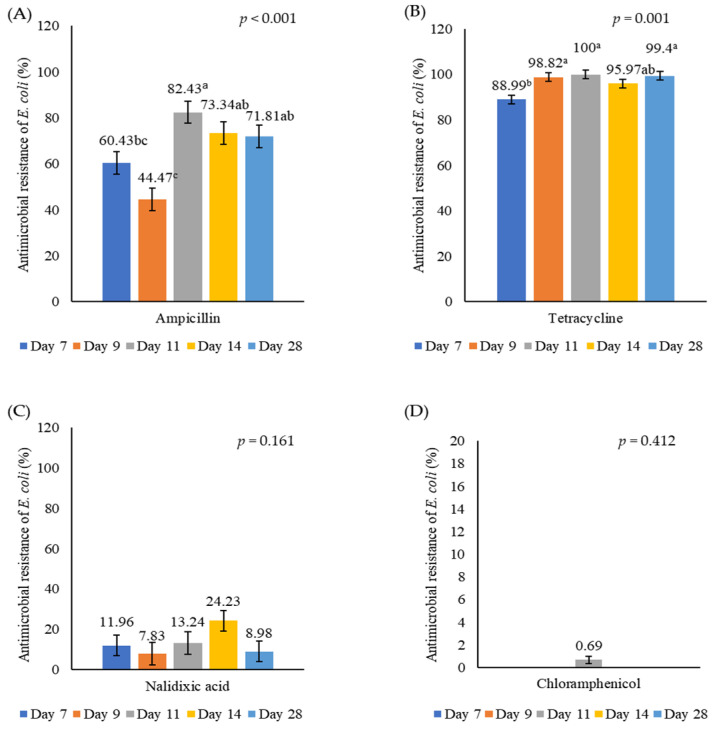
The percentage of *E. coli* isolates taken from birds of different ages (days of life) that were resistant to different antimicrobials. (**A**) Ampicillin, (**B**) tetracycline, (**C**) nalidixic acid, and (**D**) chloramphenicol. Values with different superscripts (a–c) are significantly different (*p* < 0.05).

Figure 3 illustrates the prevalence of antimicrobial resistance in *E. coli* isolated from the birds’ caeca. There was no significant effect of administering either LL or LF on the proportion of *E. coli* that were resistant to different antimicrobials. Little resistance to nalidixic acid or chloramphenicol was observed, but there was a high prevalence of resistance to both ampicillin and tetracycline. The percentage of antibiotic-resistant *E. coli* based on the birds’ age is shown in Figure 4. This increased as birds aged from 7 d (60.43%) to 11 d (82.43%) (ampicillin, *p* < 0.001) or from 7 d (88.99%) to 9 d (98.82%) (tetracycline, *p* = 0.001). There was a high resistance to AMP and TET in *E. coli* isolates taken from both the caecum and yolk sac (seven samples) on day 1. A high resistance to AMP, TET and NA was also observed on day 3, but estimating the actual prevalence of AMR on days 1 and 3 was not possible because of the overgrowth of coliforms on the parent plates. Ampicillin resistance increased during the grower phase but showed a slight decline by week 4, indicating a potential age-related reduction in resistance. In contrast, tetracycline resistance remained consistently high throughout the 4-week experimental period.

### 3.3. Identification of E. coli by WGS

**Figure 5:**
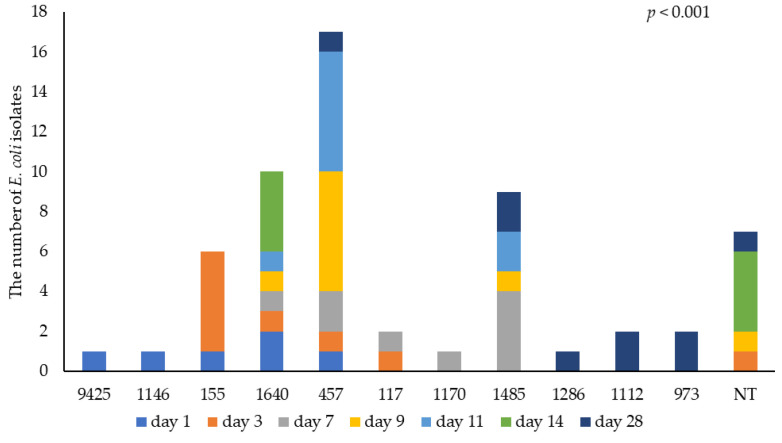
The effect of bird age (days of age) on the MLST identification of the *E. coli* isolate taken from the birds’ caecum. NT: MLST not identified.

Figure 5 illustrates the MLST of *E. coli* by bird age. A total of 11 different MLSTs were identified, with a further 7/59 isolates unknown. At 9 and 11 d of age, most isolates were MLST 457; this was the most common type (29% of isolates) throughout the study and was isolated from birds throughout their life. The second most common MLST was 1640. Another four unidentified MLSTs (NT) identified on day 14 were of the same serotype as the prevalent MLST 1640. The greatest diversity was observed during the starter phase, and new MLST 973, 1112 and 1286 were detected when birds were 28 d old (X^2^ = 122.40, *p* < 0.001). As shown in the supplementary materials, Table S1 provides a summary of the MLST and serotype classification of all *E. coli* isolates based on WGS.

### 3.4. E. coli Virulence by WGS

**Figure 6:**
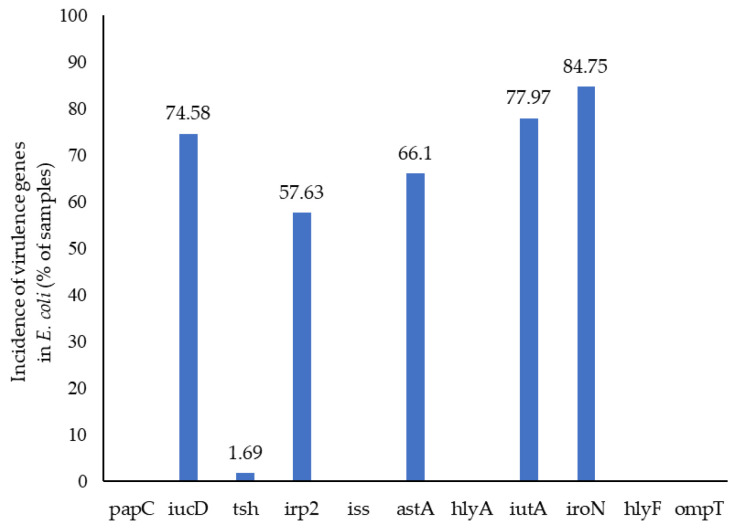
The proportion of virulence-associated genes in *E. coli* isolates taken from the caecal digesta of birds analysed by WGS.

**Figure 7:**
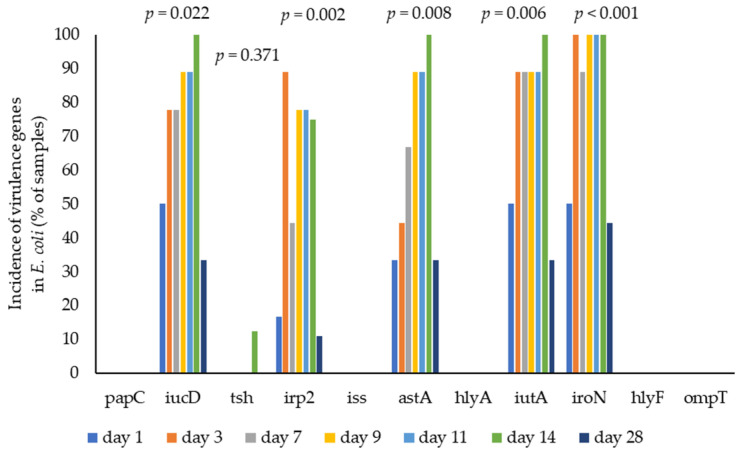
The effect of bird age on the carriage of virulence-associated genes in isolates of *E. coli* by WGS.

**Figure 8:**
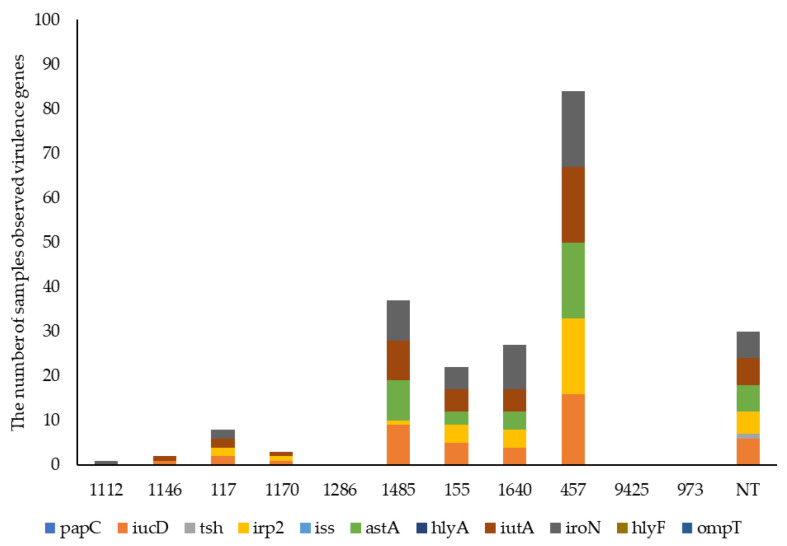
The association between MLST and virulence genes in *E. coli* isolates. NT: MLST not identified.

The proportion of virulence-associated genes in *E. coli* isolates detected by WGS analysis is presented in Figure 6. There were many and various virulence genes identified in the *E. coli* isolates, and the presence/absence of 11 virulence genes was determined. The percentages of isolates identified as carrying different genes were *iro*N (84.75%), *iut*A (77.97%), *iuc*D (74.58%), *ast*A (66.1%), *irp*2 (57.63%) and *tsh* (1.69%), with no effect of treatment. None of the isolates carried the *pap*C, *iss*, *hly*A, *hly*F or *omp*T genes. There was a low prevalence of virulence genes in seven *E. coli* isolates from the yolk sac, with only one possessing *iuc*D and *iut*A genes and two possessing the *iro*N gene. The effect of bird age on the carriage of virulence-associated genes by *E. coli* isolates, determined by WGS analysis, is shown in Figure 7. There was a high prevalence of virulence genes in *E. coli* isolates during the starter phase. The prevalence of virulence genes (*iuc*D, *irp*2, *ast*A, *iut*A and *iro*N) when determined by WGS analysis declined by 33.33%, 11.11%, 33.33%, 33.33% and 44.44%, respectively, when the birds were 28 d old (*p* = 0.022, *p* = 0.002, *p* = 0.008, *p* = 0.006 and *p* < 0.001, respectively). The association between the virulence genes and MLST is presented in Figure 8. MLST 155, 457, 1485 and 1640 possessed mainly *iuc*D, *irp*2, *ast*A, *iut*A and *iro*N during the starter phase.

## 4. Discussion

The benefits and potential of lactic acid bacteria have been demonstrated in vitro for biological activity such as antimicrobial activity, and in vivo for the improvement of immunity and effect on the microbial population in the chicken gut [18,19,20,21]. Murry et al. [22] reported that administering 0.1% botanical probiotics (Feed FreeTM) containing *L. plantarum* and *L. salivarius* increased the population of lactic acid bacteria and decreased the *Clostridium perfringens* population in the cloaca. Olnood et al. [23] reported that the population of Enterobacteria in the caeca decreased on day 35 when either unidentified *Lactobacillus* sp. or *L. salivarius* or *L. johnsonii* was administered.

However, this study observed no effect of administering either *Lactococcus lactis* or *L. fermentum* during the starter phase on the bird performance and population of coliforms and lactic acid bacteria in the caecum during the four-week experimental period. The presence of VAGs in *E. coli* colonising the gut of very young chicks can be fatal, and that may contribute to early life losses and productivity reductions in the poultry industry [24,25,26], which is why this study focused on days 1–29 rather than days 1–42.

These isolates (LAB) had both demonstrated in vitro antibacterial activity in preliminary testing, and so the lack of effect in vivo may reflect the much more complex environment of the gut. These bacteria were not administered every day, and this may also have contributed to their lack of effect. The evaluation of beneficial bacterial efficacy for the gut microbiome is very dependent on factors such as the bacterial strain used, the method and frequency of administration, and the farm environment [27,28]. It remains to be seen whether these particular LAB could manipulate microbial populations if they were administered for a longer time and under more challenging circumstances for the birds.

In this study, the high resistance to ampicillin and tetracycline was maintained throughout the birds’ life. Szmolka and Nagy [29] reported that the high prevalence of tetracycline resistance was possibly associated with the prevalence of multidrug resistance genes. A possible alternative explanation is that the resistance genes are linked to mobile genetic elements, including plasmids [30].

As observed, this finding is contrary to a previous study [14], which showed a low prevalence of AMR as birds aged. It may also reflect the change in the way that phenotypic AMR prevalence was assessed. In this study, the phenotypic AMR test was assessed by replicating plating of all cultured coliforms, while a single isolate was used for AMR by the disc diffusion assay or streaking on a plate with antibiotics in the previous study. Replicate plating for testing AMR provides a more robust estimate of the prevalence of resistance, but care is needed when conducting this methodology to prevent contamination. The plates need to be completely dried, and a rigorous aseptic technique is required. Moreover, various factors, including environmental conditions and sample size, may have influenced the outcomes of the experiment. The results of this study do not support the hypothesis that LAB administered during the starter phase reduces the prevalence of AMR *E. coli* in the starter (and grower) period. The fact that AMR *E. coli* persisted throughout this study might suggest that the birds were challenged in some way, and also that the selective advantage of the AMR coliforms persisted in the caecum throughout the birds’ lives. Further genetic analysis of AMR genes will be required to elucidate the underlying mechanisms.

One of the purposes of whole-genome sequencing is to identify the clonality of the strains and MLST and deduced O antigen type. The greatest diversity of *E. coli* strains was associated with the bird age. MLST 457 (O11:H15) was the most common type that was identified, followed by MLST 1640 (O124/O164:H25) and 1485 (O83:H42), particularly during the starter phase. However, little is known about the association between the carriage of virulence genes and MLST in *E. coli* isolates as birds age, while many studies have demonstrated the association between certain MLST *E. coli* strains and poultry colibacillosis [31,32,33]. Pires-dos-Santos et al. [34] reported that ST 95 was mostly observed in extraintestinal pathogenic *Escherichia coli* (ExPEC) isolates in both human and poultry infections. This was followed by ST 131, ST 420 and ST 648, which were related to urinary tract infections (UTIs) in humans. Colibacillosis observed in chickens aged between three and ten weeks was associated with the O1, O2 and O78 serogroups of *E. coli* [35,36,37]. In the present study, these serotypes and serogroups were not observed; instead, MLST 457, 1640, 1485, and 155 were common during the starter phase. New MLST 973, 1112 and 1286 were observed only on day 28, reflecting the diversity and evolution of *E. coli* as the birds aged.

The pathotype of *E. coli* strains can be determined by the variety of VAGs they carry. However, the determination of what characterises APEC is a matter of debate [38,39]. Johnson et al. [40] identified as minimal predictors of APEC the carriage of *iut*A, *hly*F, iss, *iro*N and *omp*T genes. In the present study, a large number of potential virulence genes were identified by WGS analysis. Genes associated with iron uptake and sequestration were very prevalent in *E. coli* isolates. This would suggest that these characteristics might confer a clear selective advantage to strains carrying these genes. Young chickens are particularly susceptible to APEC, but the declining prevalence of virulence factors in *E. coli* by day 28 might indicate that, as observed in the earlier experiment [14], commensal *E. coli* proliferates and outcompetes the APEC strains as the birds grow older. This finding supported the hypothesis that the prevalence of pathogenic coliforms decreased as birds aged. VAGs carrying *E. coli* might originate from the hatchery [41,42], and a move to hatching the egg at the production facility could reduce infection, as it would reduce the risk of infection from the hatchery and haulier.

Little is known about the interaction of bird age with the prevalence of VAGs in *E. coli* isolates from the caecum in healthy poultry, and still less is known about the role of plasmids in genetic reassortment and transfer. Identifying conditions that might reduce the spread of plasmids (and thereby the development of pathogenic strains of *E. coli*) would be a valuable research avenue. Many studies have instead comparatively researched the VAGs in *E. coli* isolates taken from either healthy chickens or those with colibacillosis [7,8]. Unsurprisingly, it was demonstrated that various specific VAGs were more prevalent in pathogenic *E. coli* compared with commensal *E. coli* [43,44,45]. Delicato, de Brito, Gaziri and Vidotto [44] observed that the *csg*A and *fim*H genes had the highest frequency in *E. coli* from both healthy and colibacillosis chickens. However, seven virulence genes (*iut*A, *tsh*, *iss*, *cva*C, *pap*C, *pap*G and *fel*A) were observed more frequently in isolates from colibacillosis birds compared with healthy chickens.

In this study, neither *pap*C, *iss*, *hly*A, *hly*F nor *omp*T was detected in any *E. coli* isolates. *iss*, *hly*F and *omp*T are plasmid-mediated and may be lost from WGS analysis during DNA preparation [46]. In addition, no isolates carried the ‘minimal predictor’ set of APEC genes (*iut*A, *hly*F, *iss*, *iro*N and *omp*T) according to the WGS analysis. It could therefore be concluded that in this study there were no APEC isolated from the caecum of any of the birds, and indeed there was no evidence that any of the birds suffered from colibacillosis. There were, however, a number of isolates that carried four or more virulence genes and which may therefore have the potential to be pathogenic to the bird, but the combinations of virulence genes carried were different from those identified in the papers referred to above. In future studies, the potential for using benign *E. coli* serotypes as a probiotic might be investigated as this might reduce the prevalence of virulent *E. coli* in young birds.

## 5. Conclusions

There was no evidence in this study that the administration of lactic acid bacteria affected the population of coliforms and lactic acid bacteria in the caecum of broilers. In addition, neither LL nor LF administration had any effect on the AMR *E. coli*. The high (phenotypic) resistance of *E. coli* to ampicillin and tetracycline was maintained throughout the life of the birds. In WGS analysis, a high prevalence of *iuc*D, *irp*2, *ast*A, *iut*A and *iro*N was observed in younger birds, but again these genes declined by day 28. MLST 457, 1640, 1485 and 155 were mostly detected in early bird life, and these serotypes had a high prevalence of *iuc*D, *irp*2, *ast*A, *iut*A and *iro*N genes. Strategies to reduce the prevalence of these potential pathogens in young birds should be developed. The young chicks’ susceptibility to putative pathogenic *E. coli* has been demonstrated. This is a potential avenue for future research, but it should be noted that the development of the gut microbiota and pathogenic bacteria are affected by a range of complex factors. Any such intervention would therefore require considerable thought and research, and would need to be considered alongside the management of other stress and environmental factors.

## Figures and Tables

**Table 1:** Ingredient and nutrient composition (as-fed basis) of chickens’ diets.

	Starter (0–14 d)	Grower/Finisher (15–28 d)
Ingredient composition (g/kg)		
Barley	40	40
Wheat (12.5% CP)	473	546
Soya bean meal (48% CP)	325	230
Rapeseed meal	42	40
Soya bean oil	50	55
L-lysine HCl	4	3
DL-methionine	3	2
L-threonine	2	2
Sodium bicarbonate	2.5	2.5
Salt	2	2.5
Limestone	10	9
Poultry vitamins/minerals ^1^	4	4
Dicalcium phosphate (QPRDC)	17.5	14
Sunflower meal (expeller)	20	45
Titanium dioxide	5	5
Nutrient composition (g/kg)		
ME (MJ/kg)	10.4	10.8
Crude protein	236.0	212.0
Total starch	287.0	312.0
Oil A (ether extract)	41.9	41.6
Sugar as sucrose	42.3	38.3
Cystine	3.7	3.6
Methionine	6.0	4.7
Lysine	13.4	12.4
Iron (mg/kg)	109	126

ME, metabolisable energy. ^1^ The vitamin/mineral premix supplied per kg of starter diets: vitamin A 13,500 IU, vitamin D_3_ 5000 IU, vitamin E 100 mg, vitamin B_1_ 3 mg, vitamin B_2_ 10 mg, vitamin B_6_ 3 mg, vitamin B_12_ 0.03 mg, nicotinic acid 60 mg, pantothenic acid 15 mg, folic acid 1.5 mg, biotin 0.25 mg, choline chloride 250 mg, Fe 20 mg, Mn 100 mg, Cu 10 mg, Zn 80 mg, I 1 mg, Se 0.25 mg, Mo 0.50 mg. The vitamin/mineral premix supplied per kg of grower/finisher diets: vitamin A 10,000 IU, vitamin D_3_ 5000 IU, vitamin E 100 mg, vitamin B_1_ 3 mg, vitamin B_2_ 10 mg, vitamin B_6_ 3 mg, vitamin B_12_ 0.03 mg, nicotinic acid 60 mg, pantothenic acid 15 mg, folic acid 1.5 mg, biotin 0.25 mg, choline chloride 250 mg, Fe 20 mg, Mn 100 mg, Cu 10 mg, Zn 80 mg, I 1 mg, Se 0.25 mg, Mo 0.50 mg.

**Table 2:** Effects of administering *Limosilactobacillus fermentum* or *Lactococcus lactis* via drinking water on growth performance in broiler chickens.

	Control	LL	LF	SEM	*p*-Value
Weight gain (days 1–14, g/bird/d)	22.51	20.06	21.26	1.23	0.39
Weight gain (days 15–28, g/bird/day)	63.8	58.3	58.2	1.79	0.061
Feed intake (days 15–28, g/bird/day)	101.7	97.9	98.0	3.12	0.623
FCR (days 15–28, g/bird/day)	1.60	1.68	1.68	0.049	0.421

LL, birds were treated with 10^7^ CFU/mL *Lactococcus lactis* (LL) in their drinking water three days a week during the starter phase; LF, birds were treated with 10^7^ CFU/mL *Limosilactobacillus fermentum* (LF) in their drinking water three days a week during the starter phase; FCR, feed conversion ratio.

## Data Availability

The original contributions presented in this study are included in the article, and further inquiries can be directed to the corresponding author.

## Supplementary Materials

The following supporting information can be downloaded at: https://www.mdpi.com/article/10.3390/ani15101353/s1, Table S1. MLST and serotype classification of *E. coli* isolates based on whole-genome sequencing. *Lactococcus lactis* (LL), *Lactobacillus fermentum* (LF).

## References

[1] Hesp A., Veldman K., van der Goot J., Mevius D., van Schaik G. (2019). Monitoring antimicrobial resistance trends in commensal *Escherichia coli* from livestock, the Netherlands, 1998 to 2016. Eurosurveillance.

[2] Majewski M., Józefiak A., Kimsa-Furdzik M., Dziubdziela L., Hudak-Nowak M., Wilczyński J., Anusz K. (2020). Antimicrobial resistance of *Escherichia coli* and *Klebsiella* spp. conventionally sampled from factory-farmed chickens–clinical submissions. Ann. Agric. Environ. Med..

[3] Kaper J.B., Nataro J.P., Mobley H.L. (2004). Pathogenic *Escherichia coli*. Nat. Rev. Microbiol..

[4] Dobrindt U. (2005). (Patho-) genomics of *Escherichia coli*. Int. J. Med. Microbiol..

[5] Nakazato G., Campos T.A.d., Stehling E.G., Brocchi M., Silveira W.D.d. (2009). Virulence factors of avian pathogenic *Escherichia coli* (APEC). Pesq. Vet. Bras..

[6] Mellata M., Dho-Moulin M., Dozois C.M., Curtiss III R., Brown P.K., Arné P., Brée A., Desautels C., Fairbrother J.M. (2003). Role of virulence factors in resistance of avian pathogenic *Escherichia coli* to serum and in pathogenicity. Infect. Immun..

[7] McPeake S., Smyth J., Ball H. (2005). Characterisation of avian pathogenic *Escherichia coli* (APEC) associated with colisepticaemia compared to faecal isolates from healthy birds. Vet. Microbiol..

[8] Paixao A., Ferreira A., Fontes M., Themudo P., Albuquerque T., Soares M., Fevereiro M., Martins L., Corrêa de Sá M. (2016). Detection of virulence-associated genes in pathogenic and commensal avian *Escherichia coli* isolates. Poult. Sci..

[9] Pitout J. (2012). Extraintestinal pathogenic *Escherichia coli*: A combination of virulence with antibiotic resistance. Front. Microbiol..

[10] Johnson T.J., Logue C.M., Johnson J.R., Kuskowski M.A., Sherwood J.S., Barnes H.J., DebRoy C., Wannemuehler Y.M., Obata-Yasuoka M., Spanjaard L. (2012). Associations between multidrug resistance, plasmid content, and virulence potential among extraintestinal pathogenic and commensal *Escherichia coli* from humans and poultry. Foodborne Pathog. Dis..

[11] Capcarova M., Weiss J., Hrncar C., Kolesarova A., Pal G. (2010). Effect of *Lactobacillus fermentum* and *Enterococcus faecium* strains on internal milieu, antioxidant status and body weight of broiler chickens. J. Anim. Physiol. Anim. Nutr..

[12] Bai S., Wu A., Ding X., Lei Y., Bai J., Zhang K., Chio J. (2013). Effects of probiotic-supplemented diets on growth performance and intestinal immune characteristics of broiler chickens. Poult. Sci..

[13] Enan G., Abdel-Shafi S., Ouda S., Negm S. (2013). Novel antibacterial activity of *Lactococcus lactis* subspecies lactis z11 isolated from zabady. Int. J. Biomed. Sci..

[14] Lee A., Aldeieg M., Woodward M., Juniper D., Rymer C. (2021). The effect of *Candida famata* and *Lactobacillus plantarum* on the number of coliforms and the antibiotic resistance and virulence of *Escherichia coli* in the gut of broilers. Animal.

[15] DEFRA (2018). Code of Practice for the Welfare of Meat Chickens and Meat Breeding Chickens.

[16] Aviagen Ross 308 Broiler Managment Handbook. http://en.aviagen.com/brands/ross/products/ross-308.

[17] Tonks A.A. (2018). Exploring the Effects of Management Strategies on the Gut Microbiome and Metabolome of Growing Broiler Chickens: An Integrated Metagenomic and Metabolomic Approach. Ph.D. Thesis.

[18] Musikasang H., Tani A., H-kittikun A., Maneerat S. (2009). Probiotic potential of lactic acid bacteria isolated from chicken gastrointestinal digestive tract. World J. Microbiol. Biotechnol..

[19] Reis J., Paula A., Casarotti S., Penna A. (2012). Lactic acid bacteria antimicrobial compounds: Characteristics and applications. Food Eng. Rev..

[20] Reuben R.C., Roy P.C., Sarkar S.L., Alam R.-U., Jahid I.K. (2019). Isolation, characterization, and assessment of lactic acid bacteria toward their selection as poultry probiotics. BMC Microbiol..

[21] Kabir S.M.L. (2009). The Role of Probiotics in the Poultry Industry. Int. J. Mol. Sci..

[22] Murry A., Hinton A., Buhr R. (2006). Effect of botanical probiotic containing Lactobacilli on growth performance and populations of bacteria in the ceca, cloaca, and carcass rinse of broiler chickens. Int. J. Poult. Sci..

[23] Olnood C.G., Beski S.S., Choct M., Iji P.A. (2015). Novel probiotics: Their effects on growth performance, gut development, microbial community and activity of broiler chickens. Anim. Nutr..

[24] Awad A.M., El-Shall N.A., Khalil D.S., El-Hack M.E.A., Swelum A.A., Mahmoud A.H., Ebaid H., Komany A., Sammour R.H., Sedeik M.E. (2020). Incidence, pathotyping, and antibiotic susceptibility of avian pathogenic *Escherichia coli* among diseased broiler chicks. Pathogens.

[25] Kemmett K., Williams N., Chaloner G., Humphrey S., Wigley P., Humphrey T. (2014). The contribution of systemic *Escherichia coli* infection to the early mortalities of commercial broiler chickens. Avian Pathol..

[26] Krishnegowda D.N., Singh B.R., Mariappan A.K., Munuswamy P., Singh K.P., Saminathan M., Ramalingam R., Chellappa M.M., Singh V., Dhama K. (2022). Molecular epidemiological studies on avian pathogenic *Escherichia coli* associated with septicemia in chickens in India. Microb. Pathog..

[27] Patterson J., Burkholder K. (2003). Application of prebiotics and probiotics in poultry production. Poult. Sci..

[28] Khan S.H., Yousaf B., Mian A.A., Rehman A., Farooq M.S. (2011). Assessing the effect of administering different probiotics in drinking water supplement on broiler performance, blood biochemistry and immune response. J. Appl. Anim. Res..

[29] Szmolka A., Nagy B. (2013). Multidrug resistant commensal *Escherichia coli* in animals and its impact for public health. Front. Microbiol..

[30] Reygaert W.C. (2018). An overview of the antimicrobial resistance mechanisms of bacteria. AIMS Microbiol..

[31] Papoušková A., Čížek A. (2020). A complex approach to a complex problem: The use of whole-genome sequencing in monitoring avian-pathogenic *Escherichia coli*—A review. Acta Vet. Brno.

[32] Gregersen R., Christensen H., Ewers C., Bisgaard M. (2010). Impact of *Escherichia coli* vaccine on parent stock mortality, first week mortality of broilers and population diversity of *E. coli* in vaccinated flocks. Avian Pathol..

[33] Ewers C., Antão E.-M., Diehl I., Philipp H.-C., Wieler L.H. (2009). Intestine and environment of the chicken as reservoirs for extraintestinal pathogenic *Escherichia coli* strains with zoonotic potential. Appl. Environ. Microbiol..

[34] Pires-dos-Santos T., Bisgaard M., Christensen H. (2013). Genetic diversity and virulence profiles of *Escherichia coli* causing salpingitis and peritonitis in broiler breeders. Vet. Microbiol..

[35] Hammoudi A., Aggad H. (2008). Antibioresistance of *Escherichia coli* strains isolated from chicken colibacillosis in Western Algeria. Turk. J. Vet. Anim. Sci..

[36] Ngeleka M., Brereton L., Brown G., Fairbrother J.M. (2002). Pathotypes of avian *Escherichia coli* as related to tsh-, pap-, pil-, and iuc-DNA sequences, and antibiotic sensitivity of isolates from internal tissues and the cloacae of broilers. Avian Dis..

[37] Dziva F., Stevens M.P. (2008). Colibacillosis in poultry: Unravelling the molecular basis of virulence of avian pathogenic *Escherichia coli* in their natural hosts. Avian Pathol..

[38] Cross A.S. (2008). What is a virulence factor?. Crit. Care.

[39] Josenhans C., Suerbaum S. (2002). The role of motility as a virulence factor in bacteria. Int. J. Med. Microbiol..

[40] Johnson T.J., Wannemuehler Y., Doetkott C., Johnson S.J., Rosenberger S.C., Nolan L.K. (2008). Identification of minimal predictors of avian pathogenic *Escherichia coli* virulence for use as a rapid diagnostic tool. J. Clin. Microbiol..

[41] Ozaki H., Matsuoka Y., Nakagawa E., Murase T. (2017). Characteristics of *Escherichia coli* isolated from broiler chickens with colibacillosis in commercial farms from a common hatchery. Poult. Sci..

[42] Zhao S., Wang C.-L., Chang S.-K., Tsai Y.-L., Chou C.-H. (2019). Characterization of *Escherichia coli* isolated from day-old chicken fluff in taiwanese hatcheries. Avian Dis..

[43] Messaili C., Messai Y., Bakour R. (2019). Virulence gene profiles, antimicrobial resistance and phylogenetic groups of fecal *Escherichia coli* strains isolated from broiler chickens in Algeria. Vet. Ital..

[44] Delicato E.R., de Brito B.G., Gaziri L.C.J., Vidotto M.C. (2003). Virulence-associated genes in *Escherichia coli* isolates from poultry with colibacillosis. Vet. Microbiol..

[45] Ghanbarpour R., Sami M., Salehi M., Ouromiei M. (2011). Phylogenetic background and virulence genes of *Escherichia coli* isolates from colisepticemic and healthy broiler chickens in Iran. Trop. Anim. Health Prod..

[46] Sarowska J., Futoma-Koloch B., Jama-Kmiecik A., Frej-Madrzak M., Ksiazczyk M., Bugla-Ploskonska G., Choroszy-Krol I. (2019). Virulence factors, prevalence and potential transmission of extraintestinal pathogenic *Escherichia coli* isolated from different sources: Recent reports. Gut Pathog..

