# Supplementary material for: Prevalence and Characterisation of Antimicrobial Resistance, Virulence Factors and Multilocus Sequence Typing (MLST) of Escherichia coli Isolated from Broiler Caeca
Source: Animals (Basel). 2025 May 8;15(10):1353. doi: 10.3390/ani15101353 (PMC12108432; doi:10.3390/ani15101353)
Supplement: Supplementary file 1 [file animals-15-01353-s001.zip › animals-3611733-supplementary.pdf]

Seq code	Treatmet	Bird ID	Stock #	description	Day	MLST	serotype
**A1**		843	CR374	digest from gut 2	1	1146	O103:H2
**A2**		925	CR375	digest from gut 3	1	9425	O45:H16
**A3**		790	CR376	digest from gut 4	1	457	O11:H25
**A4**		817	CR381	digest from gut 10	1	155	O8:H20
**A5**		855	CR384	digest from gut 14	1	no seq	no seq
**A6**		827	CR388	digest from gut 18	1	1640	O124/O164:H25
**A7**		912	CR390	digest from gut 20	1	1640	O124/O164:H25
**A8**		843	CR394	Yolk sac 2	1	-	O12:H11
**A9**		925	CR395	Yolk sac 3	1	-	O12:H11
**A10**		790	CR396	Yolk sac 4	1	-	O12:H11
**A11**		817	CR397	Yolk sac 10	1	155	O8:H20
**A12**		855	CR398	Yolk sac 14	1	no seq	no seq
**A13**		827	CR399	Yolk sac 18	1	1640	O124/O164:H25
**A14**		912	CR400	Yolk sac 20	1	1640	O124/O164:H25
**A15**	LL	824	CR402	caecum 2	3	155	O182:H21
**A16**	CON	801	CR405	caecum 5	3	1640	O124/O164:H25
**A17**	CON	899	CR407	caecum 7	3	155	O162/O101:H7
**A18**	LF	739	CR409	caecum 9	3	-	O103:H7
**A19**	LL	777	CR414	caecum 14	3	155	O8/O9/O11/O30:H19
**A20**	LF	789	CR415	caecum 15	3	155	O8:H20
**A21**	CON	761	CR419	caecum 19	3	155	O8:H51
**A22**	LF	805	CR421	caecum 21	3	117	O114:H4
**A23**	LL	714	CR423	caecum 23	3	457	O11:H25
**A24**	LL	637	CR426	caecum 2	7	457	O11:H25
**A25**	CON	707	CR429	caecum 5	7	1485	O83:H42
**A26**	CON	731	CR431	caecum 7	7	1485	O83:H42
**A27**	LF	716	CR433	caecum 9	7	1640	O124:H25
**A28**	LL	746	CR438	caecum 14	7	457	O11:H25
**A29**	LF	711	CR439	caecum 15	7	1485	O83:H42
**A30**	CON	638	CR443	caecum 19	7	1485	O83:H42
**A31**	LF	627	CR445	caecum 21	7	117	O114:H4
**A32**	LL	932	CR447	caecum 23 (1)	7	1170	-:H4
**A36**	LL	907	CR452	caecum 2	9	1485	O83:H42
**A37**	CON	905	CR455	caecum 5	9	-	O8:H51
**A38**	CON	887	CR457	caecum 7	9	457	O11:H25
**A39**	LF	811	CR459	caecum 9	9	457	O11:H25
**A40**	LL	892	CR464	caecum 14	9	457	O11:H25
**A41**	LF	713	CR465	caecum 15	9	1640	O124/O164:H25
**A42**	CON	818	CR469	caecum 19	9	457	O11:H25
**A43**	LF	896	CR471	caecum 21	9	457	O11:H25
**A44**	LL	882	CR473	caecum 23	9	457	O11:H25
**A45**	LL	851	CR476	caecum 2	11	1640	O124/O164:H25
**A46**	CON	886	CR479	caecum 5	11	457	O11:H25
**A47**	CON	870	CR481	caecum 7	11	1485	O83:H42
**A48**	LF	916	CR483	caecum 9	11	457	O11:H25
**A49**	LL	839	CR488	caecum 14	11	457	O11:H25
**A50**	LF	845	CR489	caecum 15	11	457	O11:H25
**A51**	CON	859	CR493	caecum 19	11	1485	O83:H42
**A52**	LF	763	CR495	caecum 21	11	457	O11:H25
**A53**	LL	802	CR497	caecum 23	11	457	O11:H25
**A54**	LL	766	CR500	caecum 2	14	no seq	no seq
**A55**	CON	800	CR503	caecum 5	14	-	O124/O83/O164:H25
**A56**	CON	624	CR505	caecum 7	14	1640	O124/O83/O164:H25
**A57**	LF	906	CR507	caecum 9	14	-	O124:H25
**A58**	LL	866	CR512	caecum 14	14	-	O124/O83:H25
**A59**	LF	623	CR513	caecum 15	14	1640	O124/O164/O83:H25
**A60**	CON	709	CR517	caecum 19	14	-	O124/O83/O164:H25
**A61**	LF	861	CR519	caecum 21	14	1640	O124/O83:H25
**A62**	LL	903	CR521	caecum 23	14	1640	O124/O83/O164:H25
**A63**	LL	783	CR524	caecum 2	28	-	-:H11
**A64**	CON	841	CR527	caecum 5	28	457	O11:H25
**A65**	CON	784	CR529	caecum 7	28	1485	O83:H42
**A66**	LF	848	CR531	caecum 9	28	1112	O86:H27
**A67**	LL	874	CR536	caecum 14	28	973	O11:H15
**A68**	LF	917	CR537	caecum 15	28	1112	O86:H27
**A69**	CON	842	CR541	caecum 19	28	1286	O16:H32
**A70**	LF	769	CR543	caecum 21	28	973	O11:H15
**A71**	LL	626	CR545	caecum 23	28	1485	O83:H42

Table S1. MLST and serotype classification of *E. coli* isolates based on whole-genome sequencing.
Lactococcus lactis (LL), *Lactobacillus fermentu* m (LF).
